# The Annual Commencement of the Baltimore College of Dental Surgery

**Published:** 1870-03

**Authors:** 


### EDITORIAL DEPARTMENT.

---

*The Annual Commencement of the Baltimore College of Dental Surgery.*—The *Thirtieth* Annual Commencement of the Baltimore College of Dental Surgery was held at the Concordia Opera House, on Wednesday evening, March 2nd, in the presence of a large audience. Every seat was occupied on the floor and in the galleries, by the friends of the graduates, the majority of whom were ladies. At 8.30 P. M, the members of the graduating class preceded by the Faculty and followed by a number of distinguished guests, among whom were representatives from the two Medical Colleges of Baltimore, entered upon the stage. After some choice selections of music, executed by the Blues Band under the direction of Prof. Holland, the exercises were opened with prayer bv the Rev. J. B. Fitzpatrick of Virginia.

Professor F. J. S. Gorgas, the Dean of the Faculty, then read extracts from the act of incorporation granted by the General Assembly of Maryland in 1839, empowering the Faculty to confer the degree of Doctor of Dental Surgery on all students who shall have complied with the rules of the institution and then announced the names of the graduates. After music, the graduates came forward as their names were announced, and were presented with their diplomas by the dean, who, by virtue of the authority committed to the Faculty, conferred upon them the degree of "Doctor of Dental Surgery," with all the rights and privileges pertaining thereto. The following is the list of graduates for 1870 :

Louis Augspath,............................................*Russia.*
William Robert Ballard, Jr., D.D.S.............*England.*
William Henry Bennett,...........................*Tennessee.*
Clinton Thomas Brockett,............................*Maryland.*
Benjamin Holliday Catching,..................*Mississippi.*
Alexander Dunnington Cobey,...................*Maryland.*
Abraham F. Cox,.......................................*Virginia.*
John Henry Coyle,......................................*Georgia.*
Kurwin L. Eisenhart,...........................*Pennsylvania.*
Edward Stabler Fawcett,................................*Virginia.*
Hillary Edgar Hardey, ..........................*Maryland.*
Louis Summerfield Ledbetter,.......................*Georgia.*
James Henry Ludwig, M. D.,.....................*Maryland,*
Jonathan Magruder,...................................*Maryland.*
John William Meng,...................................*Missouri.*
Eber Rice Perrow,......................................*Virginia.*
Oscar Ernst Moritz Salomon,................*North Germany.*
Thomas James Speck,................................*Tennessee.*
David Franklin, Swengel,......................*Pennsylvania,*
Henry Grove Ulrich,...........................*Pennsylvania.*
Andrew Park White,................................*Tennessee.*
John Thompson Wilson,................................*Virginia.*
Thruston Wolfe,.......................................*Virginia.*
W. Tryon Yarbrough,...........................*Mississippi.*

The Valedictory Address was then delivered by Dr. M. J. DeRosset, Professor of Chemistry, who impressed upon the graduates the importance of continued study, gave some excellent advice to those about to enter upon a professional life, and asked them to uphold the honor of their Alma Mater at all times. This address was followed by one on behalf of the graduating class, delivered by Dr. B. H. Catching, of Mississippi, a member of the class, which was well written and also well delivered. The graduates were the recipients of a large number of handsome boquets from their lady friends, and every thing passed off in the most happy and agreeable manner. The exercises of the Commencement were concluded by a benediction, and thus another session of this *pioneer* of Dental Colleges ended.

